# Longitudinal modeling of human neuronal aging identifies RCAN1-TFEB pathway contributing to neurodegeneration of Huntington’s disease

**DOI:** 10.21203/rs.3.rs-2815300/v1

**Published:** 2023-05-09

**Authors:** Seong Won Lee, Young Mi Oh, Matheus B. Victor, Ilya Strunilin, Shawei Chen, Sonika Dahiya, Roland E. Dolle, Stephen C. Pak, Gary A. Silverman, David H. Perlmutter, Andrew S. Yoo

**Affiliations:** 1 Department of Developmental Biology, Washington University School of Medicine, St. Louis, MO 63110, USA.; 2 Center of Regenerative Medicine, Washington University School of Medicine, St. Louis, MO 63110, USA.; 3 Department of Brain and Cognitive Sciences, Massachusetts Institute of Technology, Cambridge, MA 02139, USA.; 4 Department of Pathology and Immunology, Washington University School of Medicine, St. Louis, MO 63110; 5 Department of Biochemistry, Washington University School of Medicine, St. Louis, MO 63110, USA.; 6 Department of Pediatrics, Washington University School of Medicine, St. Louis, MO 63110, USA.

## Abstract

Aging is a common risk factor in neurodegenerative disorders and the ability to investigate aging of neurons in an isogenic background would facilitate discovering the interplay between neuronal aging and onset of neurodegeneration. Here, we perform direct neuronal reprogramming of longitudinally collected human fibroblasts to reveal genetic pathways altered at different ages. Comparative transcriptome analysis of longitudinally aged striatal medium spiny neurons (MSNs), a primary neuronal subtype affected in Huntington’s disease (HD), identified pathways associated with RCAN1, a negative regulator of calcineurin. Notably, RCAN1 undergoes age-dependent increase at the protein level detected in reprogrammed MSNs as well as in human postmortem striatum. In patient-derived MSNs of adult-onset HD (HD-MSNs), counteracting *RCAN1* by gene knockdown (KD) rescued HD-MSNs from degeneration. The protective effect of *RCAN1* KD was associated with enhanced chromatin accessibility of genes involved in longevity and autophagy, mediated through enhanced calcineurin activity, which in turn dephosphorylates and promotes nuclear localization of TFEB transcription factor. Furthermore, we reveal that G2-115 compound, an analog of glibenclamide with autophagy-enhancing activities, reduces the RCAN1-Calcineurin interaction, phenocopying the effect of *RCAN1* KD. Our results demonstrate that RCAN1 is a potential genetic or pharmacological target whose reduction-of-function increases neuronal resilience to neurodegeneration in HD through chromatin reconfiguration.

Aging is a major risk factor in most forms of neurodegenerative diseases and age-related changes affect many cellular processes leading to disease pathology^1-5^. While longitudinal studies in human individuals have been performed to assess the risk of aging in late-onset disorders, it is unfeasible to model this aging process with longitudinally collected human neurons. Therefore, there is a need to establish a human neuron platform that allows for studies of aging effects in an isogenic background. For generating aged human neurons, direct fate conversion of adult fibroblasts to neurons has been shown to propagate chronological age-related characteristics such as epigenetic cellular age stored in starting fibroblasts, thereby generating neurons that mimic the epigenetic age of fibroblast donors^6^. For producing disease-relevant neuronal subtypes, ectopic expression of neurogenic microRNAs, miR-9/9* and miR-124 (miR-9/9*-124), in human fibroblasts induce chromatin reconfiguration landscape upon which subtype-defining transcription factors (TFs) guide the conversion to specific types of neurons^7,8^. As such, striatal medium spiny neurons (MSNs) directly reprogrammed from fibroblasts of Huntington’s disease (HD) patients (HD-MSNs) recapitulate hallmarks of adult-onset HD pathologies including mutant HTT (mHTT) aggregation and neurodegeneration^9-11^. Thus, directly reprogrammed human MSNs serve as a patient-derived neuron model that captures age-dependent adult-onset degenerative pathology of HD^6,9-11^.

Directly reprogrammed MSNs retain age-associated epigenetic signatures stored in starting fibroblasts^6^. In this study, we utilized MSNs directly reprogrammed from isogenic, longitudinally collected fibroblasts to identify age-associated transcriptome changes in MSNs. We then applied these findings in HD by testing whether the age-associated genes can be perturbed to protect HD-MSNs from neurodegeneration. Through comparative transcriptome analyses between reprogrammed MSNs from longitudinal young and old ages, we reveal age-associated increase in RCAN1 protein both in reprogrammed MSNs and postmortem human striatum. RCAN1 is an inhibitory interactor of calcineurin (CaN)^12,13^, a calcium- and calmodulin-dependent serine/threonine phosphatase, which in turn regulates phosphorylation and nuclear localization of target transcription factors (TFs)^14-16^. In human brains, RCAN1 is widely expressed in various cell types within the nervous system but most highly expressed in neurons of elderly individuals^17-19^. Whether RCAN1 would directly contribute to the age-dependent onset of neurodegeneration remains to be carefully dissected in a human neuron model of neurodegeneration. Interestingly, *RCAN1* resides in chromosome 21 where the increased gene dosage in trisomy 21 Down syndrome could be linked to increased susceptibility to Alzheimer’s disease^20-24^.

In this study, we show that dampening the age-associated increase in RCAN1 protects HD patient-derived MSNs from neurodegeneration. We provide a series of evidence that this protective effect of *RCAN1* KD results from the enhanced CaN activity, leading to dephosphorylation and nuclear localization of TFEB, an autophagy regulator^25-27^ and increased accessibility of chromatin regions that harbor TFEB binding sites. Moreover, we reveal that one of the G2 analog series^28^ is a small molecule that can phenocopy the protective effect of *RCAN1* KD by reducing the RCAN1-CaN interaction and, in turn, phosphorylation of TFEB. Collectively, our study highlights RCAN1 as an effective genetic or pharmacological target that can confer neuronal resilience against the age-associated neurodegeneration of HD.

## Results

### Neuronal conversion of longitudinally collected human adult fibroblasts and transcriptome analysis

**Fig. 1 ∣:**
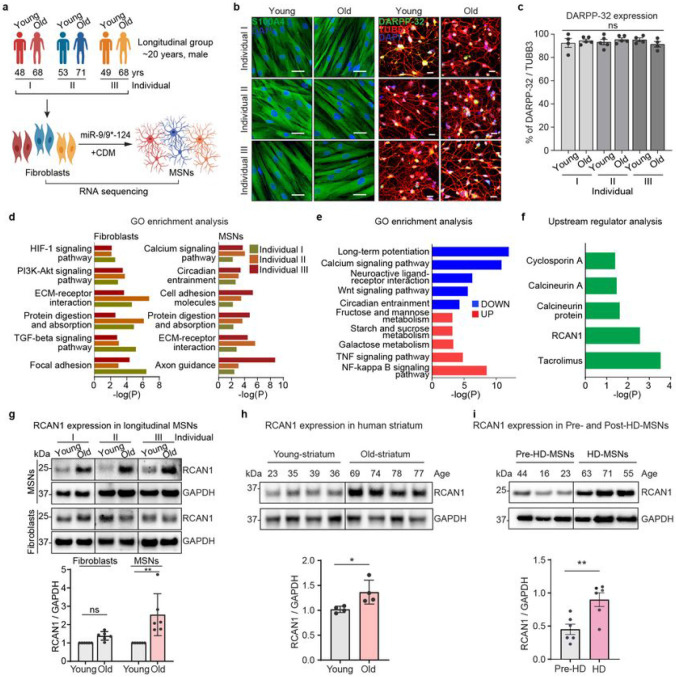
Identification of RCAN1 as an age-associated factor in reprogrammed MSNs from longitudinally collected fibroblasts **a,** Experimental scheme of RNA-sequencing in fibroblasts and reprogrammed MSNs (young and old) from three independent longitudinal groups (individual I, II, III). MSNs were reprogrammed by overexpressing miR-9/9* and miR-124 (miR-9/9*-124) as well as MSN-defining transcription factors, CTIP2, DLX1, DLX2, and MYT1L (CDM). **b**, Representative images of fibroblasts of young and old longitudinal groups marked by S100A4 (left) and reprogrammed MSNs marked by DARPP-32 from three individuals. **c**, Quantification of DARPP-32 positive cells from reprogrammed MSNs from all samples (n=4~5 replicates per sample from 6 individuals). An average of 300 cells were counted from four or more randomly chosen fields. Scale bars represent 20 μm. **d**, Gene Ontology (GO) enrichment analysis of all DEGs in two replicates of old-fibroblasts (left) and old-MSNs (right) from three independent individuals (FDR<0.05, ∣FC∣≥1.5). **e**, GO enrichment analysis of up-/down-regulated genes commonly manifested in old-MSNs compared to young-MSNs (FDR<0.05, ∣FC∣≥1.5). **f**, Upstream regulator analysis of up-/down-regulated genes in old-MSNs in (**e**). **g**, Representative Immunoblotting (top) and quantification (bottom) of RCAN1 in six longitudinal MSNs and six fibroblasts (young and old) from three independent individuals (n=6 replicates). The quantification is normalized to values from young samples per line. **h**. Representative immunoblotting (top) and quantification (bottom) of RCAN1 expression in eight human striatum samples aged 23, 35, 39, 36 (young) and 69, 74, 78, 77 (old) years old (n=8 individuals). **i**, Representative immunoblotting (top) and quantification (bottom) of RCAN1 expression in three reprogrammed MSNs from presymptomatic patients aged 44, 16, 23 years old (pre-HD-MSN: Pre-HD.42, Pre-HD.45, Pre-HD.40/50) and three reprogrammed MSNs from symptomatic patients aged 63, 71, 55 years old (HD-MSN: HD.47, HD.40, HD.45) (n=6 replicates). Statistical significance was determined using one-way ANOVA (**c**) and unpaired t-test (**g**,**h**,**i**); **p<0.01, *p<0.05, ns, not significant and mean±s.e.m. The sample size (n) corresponds to the number of biological replicates.

We investigated age-related differences in reprogrammed MSNs from longitudinally collected fibroblasts from three independent healthy individuals and carried out comparative transcriptome analysis between the age groups (Fig. 1a). We designate fibroblasts initially collected during middle age as “young” and samples subsequently collected approximately 20 years later from three independent individuals as “old” groups (Coriell NINDS and NIGMS Repositories: AG10049 (48 years), AG16030 (68 years); AG10047 (53 years), AG14048 (71 years); AG04456 (49 years), AG14251 (68 years) (Extended Data Fig. 1a, and Supplementary Table 1). We validated the MSN identity of reprogrammed neurons from all groups by assessing the expression of an MSN marker (DARPP-32) (Fig. 1b-c and Extended Data Fig. 1b). RNA-seq analysis in both longitudinally collected fibroblasts and corresponding reprogrammed MSNs revealed differentially expressed genes (DEGs) between young and old samples (FDR<0.05, ∣FC∣≥1.5) (Extended Data Fig. 1c-d). Genes up- or down-regulated in both old fibroblast and old MSNs were commonly enriched in age-associated pathways such as ECM-receptor interaction, protein digestion, cell adhesion molecules, and focal adhesion (Fig. 1d). Interestingly, down-regulated genes commonly manifested in old-MSNs over young-MSNs (FDR<0.05, ∣FC∣≥1.5) were uniquely enriched in calcium signaling pathway, yet not detected in fibroblasts, suggesting MSN-specific alteration in calcium signaling pathway (Fig. 1e and Extended Data Fig. 1e).

Ingenuity pathway analysis (IPA) revealed upstream effectors of DEGs in old-fibroblasts or old-MSNs (FDR<0.05, ∣FC∣≥1.5) (Extended Data Fig. 2a-b). Among these, RCAN1 was identified as an MSN-specific upstream effector of calcium signaling pathway as well as inhibitors of CaN (Tacrolimus and Cyclosporin A) (Fig. 1f), suggesting that MSNs reprogrammed from older individuals behave as if CaN function has been compromised.

### Age-associated upregulation of RCAN1 in MSNs

To further investigate whether RCAN1 expression may be related to aging, we assessed the RCAN1 expression between longitudinal fibroblasts and corresponding reprogrammed MSNs. As assessed by immunoblots, RCAN1 protein was expressed at a higher level in old-MSNs compared to the younger samples, whereas this difference was not detected between young- and old-fibroblasts (Fig. 1g). This MSN-enriched upregulation of RCAN1 with aging was consistent in reprogrammed MSNs from other multiple individuals (~30 years of age difference) (Extended Data Fig. 2c) and human stratum (~40 years of age difference) (Fig. 1h) between young and old samples at the protein level, but not in transcripts (Extended Data Fig. 2d). Moreover, RCAN1 expression was significantly increased in HD-MSNs from older symptomatic patients compared to HD-MSNs from younger presymptomatic patients (pre-HD-MSNs) (~35 years age difference) (Fig. 1i), suggesting the global age-associated upregulation of RCAN1 in MSNs. Altogether, our results indicate that RCAN1 is an age-associated factor whose protein expression undergoes upregulation in aged-MSNs.

### Validation of RCAN1 as a disease modifier gene in HD

Genome-wide association studies have identified Genetic Modifiers of HD (GeM-HD) comprised of polymorphic gene variants associated with accelerated or delayed onset of HD^29^. These genes were discovered as modifiers that can affect the age of symptomatic onset. Interestingly, *RCAN1* is also included in the list of candidate HD-modifier genes. Thus, as a parallel investigation, we knocked down 246 candidate genes to identify genes whose reduction-of-function would protect HD-MSNs from degeneration, thereby genes that contribute to HD-MSN degeneration (Extended Data Fig. 3a and Supplementary Table 2). Reprogrammed HD-MSNs (Extended Data Fig. 3b) were cultured in 96-well plates to be assayed for neuronal death using Sytox-Green as previously described (approximately 3,000 cells counted per well) (Extended Data Fig. 3c)^9,10^. For this assay, we first used HD-MSN from the GM04194 line (CAG repeat size 46; HD.46) which showed a two-fold increase in cell death at around 50% compared to Control (Ctrl)-MSNs from the healthy individual (GM02171) (Extended Data Fig. 3d). We then added lentivirus carrying gene-specific shRNAs to HD-MSN and the average cell death level for each gene KD was compared to the average level with scrambled control shRNA (shCtrl) (Extended Data Fig. 3e). Interestingly, in this unbiased testing, we also identified *RCAN1* whose KD led to the most significant reduction in neuronal death compared to other identified genes, *RTCA* and *UBE2D4* (pink zone = plus or minus 10% of healthy control level). This protective effect was further validated in HD-MSNs from other independent HD patients (Extended Data Fig. 3f). We also tested if KD of the identified genes would lower mHTT aggregation and found that among the genes tested, only *RCAN1* KD significantly decreased the amount of mHTT inclusion bodies (Extended Data Fig. 3g).

**Fig. 2 ∣:**
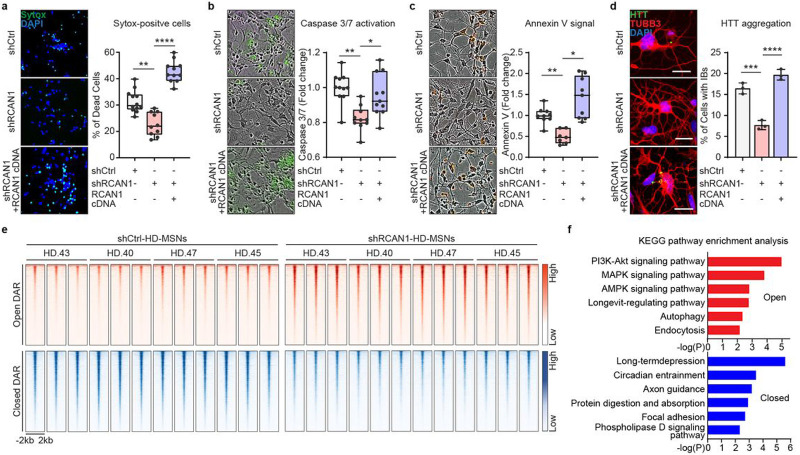
*RCAN1* KD protects HD-MSNs from degeneration and induces chromatin accessibility changes. **a-c**, Representative images (left) and quantification (right) of Sytox-positive cells (**a**), Caspase 3/7 activation (green) (**b**), and Annexin V signal (red) (**c**) in three independent HD-MSNs (HD.40, HD.43, HD.47, n=10~12, independent reprogramming experiments) transduced with shControl (shCtrl), shRCAN1, or RCAN1 cDNA. **d**, Representative images (left) and quantification (right) of cells with HTT inclusion bodies (IBs) in three independent HD-MSNs (HD.40, HD.43, HD.47, n=3) transduced with shCtrl, shRCAN1, or RCAN1. Cells were immunostained with anti-HTT and TUBB3 antibodies. An average of 120 cells per each were counted from three or more randomly chosen fields. Scale bars represent 20 μm. **e** and **f**, Analysis of ATAC-sequencing in four independent HD-MSNs (HD.43, HD.40, HD.47, HD.45) transduced with shCtrl or shRCAN1. Heatmaps of signal intensity (**e**) in chromatin peaks (FDR<0.05, ∣FC∣≥1.5) of open and closed DARs in shRCAN1-HD-MSNs compared to shCtrl-HD-MSNs. KEGG pathway enrichment analysis (**f**) of genes associated with open (top) and closed (bottom) DARs in shRCAN1-HD-MSNs. Statistical significance was determined using one-way ANOVA (**a**-**d**); ****p<0.0001, ***p<0.001, **p<0.01, *p<0.05, and mean±s.e.m. The sample size (n) corresponds to the number of biological replicates (**a-d**).

To confirm the specificity of shRCAN1 for HD survival, we prolonged RCAN1 expression by overexpressing *RCAN1* cDNA in HD-MSNs in the presence of shRCAN1 (Extended Data Fig. 4a). Continuous RCAN1 expression reversed the neuroprotective effect of shRCAN1 in HD-MSNs from multiple patients (Fig. 2a). Furthermore, since caspase activation signals have been detected in HD patient brains^30-36^, we also assessed Caspase 3/7 activation and Annexin V signal (an apoptotic marker via its ability to bind to phosphatidylserine on the extracellular surface) in HD-MSNs as previously described^10^. *RCAN1* KD significantly reduced Caspase activation and Annexin V signals while the rescuing effect of shRCAN1 was abolished by RCAN1 cDNA (Fig. 2b-c). Moreover, the clearance of HTT aggregation following *RCAN1* KD was also reversed by overexpressing RCAN1 in the presence of shRCAN1 (Fig. 2d). Therefore, our results overall indicate that RCAN1 is an age-associated disease modifier whose KD leads to clearance of mHTT aggregation and neuroprotection of HD-MSNs.

### Neurodegeneration via *RCAN1* KD is associated with changes in chromatin accessibility.

RCAN1 primarily functions to inhibit its interacting partner, CaN, calcium- and calmodulin-dependent protein serine/threonine phosphatase, which in turn regulates phosphorylation of target transcription factors (TFs)^14-16^. Due to this potential link between *RCAN1* KD and changes in TF activities, we investigated whether *RCAN1* KD would lead to changes in chromatin accessibilities in HD-MSNs by performing comparative Omni-ATAC-seq^37^ analyses between shCtrl- and shRCAN1-expressing HD-MSNs from multiple HD samples (Fig. 2e) (ND30013 (HD.43), ND33947 (HD.40), GM04198 (HD.47), GM04230 (HD.45), three biological replicates per each independent line) (Supplementary Table 1). Of the total number of 102,747 peaks detected across samples, we identified 15,767 differentially accessible regions (DARs) (FDR<0.05, ∣FC∣≥1.5) between shCtrl- and shRCAN1-HD-MSNs (15.345 % of the total peaks). Of the total DARs, 6,050 DARs corresponded to chromatin regions that became more accessible (open) and 9,717 DARs less accessible (closed) in shRCAN1-HD-MSNs compared to shCtrl-HD-MSNs. Focusing on DARs ± 2 kb around the transcription start site (TSS), we identified 505 genes with increased and 1173 genes with decreased ATAC signals with shRCAN1 compared to shCtrl (FDR<0.05, ∣FC∣≥1.5) (Fig. 2e).

KEGG pathway enrichment analysis revealed that genes associated with DARs opened by shRCAN1 in HD-MSNs were enriched with longevity-regulating pathway, PI3K-AKT/ MAPK/AMPK signaling pathway, autophagy, and endocytosis, suggesting that *RCAN1* KD led to chromatin changes proximal to genes associated with aging in HD-MSNs (Fig. 2f top). Interestingly, the role of autophagy in clearing mHTT aggregates and neuroprotection was previously shown by the discovery of miR-29b-3p-STAT3 axis, Beclin1, and autophagy-related FYVE protein (ALFY) that modifies the amount of mHTT aggregation^10,38,39^. Genes associated with closed DARs in shRCAN1-HD-MSNs were, however, enriched in other pathways including long-term depression, circadian entrainment, axon guidance, protein digestion and absorption, focal adhesion, and phospholipase D signaling pathway (Fig. 2f bottom). Altogether, these results demonstrate that the protective effect of RCNA1 KD is accompanied by increased chromatin accessibility to genes involved in longevity and autophagy.

### Abolition of RCNA1 KD-mediated neuroprotection by CaN inhibition

**Fig. 3 ∣:**
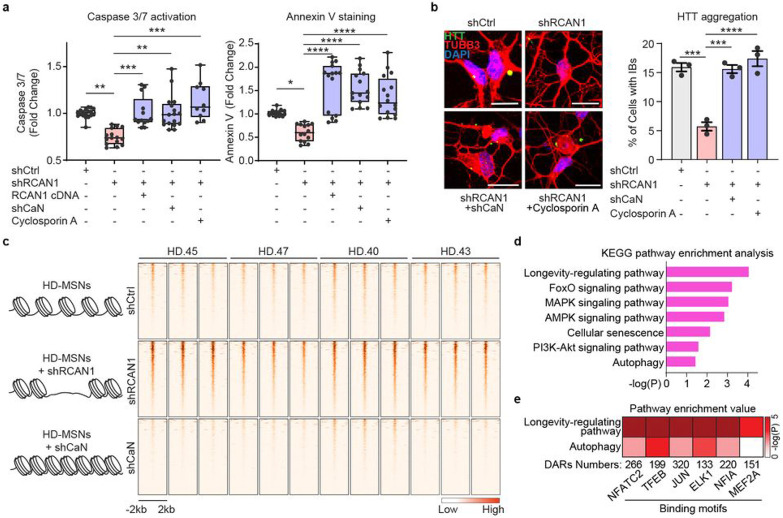
*RCAN1* KD and *CaN* KD-induced chromatin changes **a**, Quantification of caspase 3/7 activation (left) and annexin V signal (right) in four independent HD-MSNs (HD43, HD40, HD47, HD45, n=10~18) transduced with shCtrl, shRCAN1, RCAN1, or shCalcineurin (shCaN). Cells were also treated with 10 μM of Cyclosporin A, a CaN inhibitor. **b**, Representative images (left) and quantification (right) of cells with HTT inclusion bodies (IBs) in three independent HD-MSNs (HD.43, HD.40, HD.47, n=3) transduced with shCtrl, shRCAN1, or shCaN. Cells were treated with 10 μM of Cyclosporin A, a CaN inhibitor. Cells were immunostained with anti-HTT and TUBB3 antibodies. An average of 117 cells of each were counted from three or more randomly chosen fields. Scale bars represent 20 μm. **c-e**, Analysis of ATAC-sequencing from four independent HD-MSNs (HD43, HD40, HD47, HD45, three replicates each) transduced with shCtrl (control), shRCAN1 (rescuing), or shCaN (detrimental). Heatmaps (**c**) of signal intensity in overlapping chromatin peaks of open DAR (FDR<0.05, FC≥1.5) in shRCAN1-HD-MSNs and closed DAR (FDR<0.05, FC≤−1.5) in shCaN-HD-MSNs compared to shCtrl-HD-MSNs. KEGG pathway enrichment analysis (**d**) and pathway enrichment analysis (**e**) of genes associated with open DARs in shRCAN1-HD-MSNs and closed DARs in shCaN-HD-MSNs in (**c**). Statistical significance was determined using one-way ANOVA in (**a,b**); ****p<0.0001, ***p<0.001, **p<0.01, *p<0.05, and mean±s.e.m. Each dot represents one individual’s reprogrammed HD-MSNs (**a**,**b**). The sample size (n) corresponds to the number of biological replicates (**a**,**b**).

Next, we asked whether *RCAN1* KD-induced changes in chromatin would occur through CaN. Because RCAN1 normally inhibits CaN, we first tested whether inhibiting *CaN* simultaneously while knocking down *RCAN1* would revert HD-MSNs to degeneration. *RCAN1* KD decreased Caspase 3/7 activation and Annexin V signals in HD-MSNs, whereas inhibiting CaN using shRNA specific for Calcineurin A (shCaN) or cyclosporin A, a well-established inhibitor of CaN^40^, abolished the neuroprotective effect of *RCAN1* KD (Fig. 3a). Additionally, shRCAN1 failed to reduce HTT inclusion bodies when *RCAN1* KD-HD-MSNs were treated with shCaN or cyclosporine A (Fig. 3b). These results demonstrate that the protective effect of *RCAN1* KD occurs through CaN activity in HD-MSNs.

We then leveraged the finding that *CaN* KD reverses the neuroprotection by *RCAN1* KD to infer DARs opened by *RCAN1* KD that are instead closed in response to *CaN* KD. We performed a comparative ATAC-seq analysis between shRCAN1-HD-MSNs (rescuing condition) and shCaN-HD-MSNs (non-rescuing condition) where DARs in opposite directions were defined by comparisons to shCtrl-HD-MSNs (Fig. 3c and Extended Data Fig. 4b-c). 467 ATAC peaks that opened with *RCAN1* KD compared to shCtrl were overlapped with chromatin regions that instead closed with shCaN compared to shCtrl were identified (FDR<0.05, ∣FC∣≥1.5) (Figure 3c). These overlapping DARs ± 2 kb around TSS identified 286 genes whose pathway analysis identified terms associated with longevity-regulating pathway, FoxO signaling, cellular senescence, and autophagy (Fig. 3d). Therefore, these results demonstrate that *RCAN1* KD-induced neuroprotection through CaN is accompanied by chromatin changes proximal to genes involved in aging and autophagy.

### *RCAN1* KD opens chromatin regions enriched with TFEB binding sites.

CaN, a Ser/Thr phosphatase, has been shown to partner with various TFs (NFATC2, TFEB, JUN, ELK1, NF1A, and MEF2A)^41-46^ to regulate their phosphorylation and activities^14-16^. We searched sequence motifs using the JASPAR transcription factor database^47^ within DARs corresponding to regions that became more accessible with shRCAN1 (FDR<0.05, FC≥1.5) and closed with shCaN (FDR<0.05, FC≤−1.5), and found that binding sites for TFs including NFATC2, TFEB, JUN, ELK1, NF1A, and MEF2A enriched in the opposite DARs (Fig. 3e). We analyzed the pathway enrichment value (−log(P)) from KEGG pathway enrichment analysis for associated genes and the DAR number. Among the TF sites within the identified DARs, TFEB binding site was most significantly enriched with genes associated with both longevity and autophagy (Fig. 3e), suggesting TFEB as a critical TF associated with the protective role of *RCAN1* KD in HD-MSNs.

### *RCAN1* KD enhances TFEB function by promoting its nuclear localization in HD-MSNs.

**Fig. 4 ∣:**
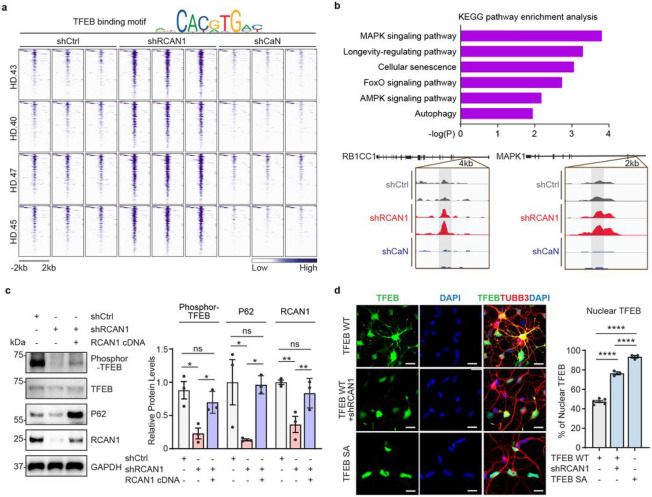
Enhancing TFEB function by *RCAN1* KD via its nuclear localization **a**, Heatmap representation of open DARs with shRCAN1 (rescuing) and closed DARs with shCaN (detrimental) harboring TFEB binding motifs, compared to shCtrl Motif analysis from ATAC-sequencing was from four independent HD-MSNs (HD.43, HD.40, HD.47, HD.45, three replicates each) (FDR<0.05, FC≥−1.5). Top legend depicts representative motifs for TFEB binding sites. **b**, KEGG pathway enrichment analysis (top) of TFEB-binding motif containing genes associated with DARs in (**a**). Integrative Genomics Viewer (IGV) snapshots (bottom) showing peaks enriched in shRCAN1-HD-MSNs (red) and reduced in shCaN-HD-MSNs (blue) within *RB1CC1* and *MAPK1* in comparison to shCtrl (grey). **c,** Representative Immunoblotting (left) and quantification (right) of the expression of phosphor-TFEB (Ser142) from three independent HD-MSNs (HD.43, HD.40, HD.47, n=3) transduced with shCtrl, shRCAN1, or RCAN1. **d,** Representative image (left) and quantification (right) of nuclear TFEB from three-independent HD-MSNs (HD.43, HD.40, HD.47, n=3~6) transduced with TFEB wildtype (WT), shRCAN1, or TFEB phosphor-mutant (S142/211A, SA). Cells were immunostained with anti-TFEB and TUBB3 antibodies. An average of 130 cells per each were counted from three or more randomly chosen fields. Scale bars represent 20 μm. Statistical significance was determined using one-way ANOVA in (**c,d)**; ****p<0.0001, **p<0.01, *p<0.05, ns: not significant, and mean±s.e.m. Each dot represents one individual’s reprogrammed HD-MSNs (**c**,**d**). The sample size (n) corresponds to the number of biological replicates (**c**,**d**).

We further confirmed the TFEB binding site enrichment by separately extracting the DARs containing TFEB binding sites corresponding to regions that became more accessible in shRCAN1-HD-MSNs (FDR<0.05, FC≥1.5) and closed in shCaN-HD-MSNs (FDR<0.05, FC≤−1.5) compared to shCtrl-HD-MSNs (Fig. 4a). Genes proximal to these oppositely accessible DARs containing TFEB binding sites were enriched with longevity and autophagy pathways (Fig. 4b top). Some of these genes include *RB1CC1,* an autophagy inducer^48,49^, and *MAPK1,* whose function has been shown to decline with brain aging^50,51^ (Fig. 4b bottom).

TFEB is known as a regulator of lysosomal biogenesis and autophagy^25-27^ which may also regulate longevity^52-57^. Phosphorylation keeps TFEB localization in the cytoplasm whereas its dephosphorylation allows TFEB shuttling into the nucleus^26,58,59^. Since RCAN1 inhibits CaN function and CaN has been shown to dephosphorylate TFEB^41^, we tested whether *RCAN1* KD would lead to TFEB dephosphorylation and nuclear localization in HD-MSNs. *RCAN1* KD reduced the level of phosphorylated TFEB, which was reversed by overexpressing RCAN1 as assessed by immunoblots in HD-MSNs from multiple HD patients (Fig. 4c). Also, while expressing exogenous TFEB led to the localization of TFEB in both cytoplasm and nucleus, *RCAN1* KD significantly increased nuclear localization of TFEB, which was mimicked by phosphor-mutant (S142/211A) TFEB containing mutations at the serine residue S142 and S211 (CaN’s dephosphorylation sites in TFEB) to alanine^26,41,60^ (Fig. 4d and Extended Data Fig. 5a). Our results thus indicate that *RCAN1* KD enhances TFEB activity by promoting its nuclear localization.

### *RCAN1* KD promotes neuronal resilience against degeneration via enhancing TFEB function.

**Fig. 5 ∣:**
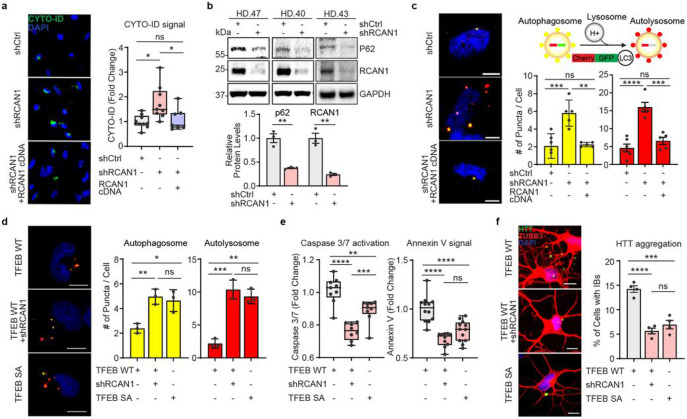
*RCAN1* KD promotes neuronal resilience through enhancing TFEB nuclear localization. **a**, Representative images (left) and quantification (right) of CYTO-ID-positive cells from three independent HD-MSNs (HD.43, HD.40, HD.47, n=7~9) transduced with shCtrl, shRCAN1, or RCAN1. **b**, Immunoblotting (top) and quantification (bottom) of the expression of p62 and RCAN1 from three independent HD-MSNs (HD.43, HD.40, HD.47, n=3) transduced with shCtrl or shRCAN1. **c**, Autophagic flux measurements using tandem monomeric mCherry-GFP-LC3 (right top). Representative image (left) and quantification (right bottom) of autophagosome and autolysosome from cells having reporter signal puncta from three independent HD-MSNs (HD.43, HD.40, HD.47, n=5~6) transduced with shCtrl, shRCAN1, or RCAN1. **d**, Representative image (left) and quantification (right) of autophagosome and autolysosome from cells having puncta from three independent HD-MSNs (HD.43, HD.40, HD.47, n=3) transduced with TFEB Wildtype (WT), shRCAN1, or TFEB Phospho-mutant (SA, S142/211A). **e**, Quantification of Caspase 3/7 activation (left) and Annexin V signal (right) from three independent HD-MSNs (HD.40, HD.43, HD.47, n=8~12) transduced with TFEB WT, shRCAN1 or TFEB SA. **f**, Representative images (left) and quantification (right) of HTT inclusion bodies (IBs) from four independent HD-MSNs (HD.47, HD.40, HD.43, HD.45, n=4) transduced with TFEB WT, shRCAN1, or TFEB SA. Cells were immunostained with anti-HTT and TUBB3 antibodies. An average of 120 cells per each were counted from three or more randomly chosen fields. Scale bars represent 10 μm (**c**,**d**,**f**). Statistical significance was determined using one-way ANOVA (**a**,**c**,**d**,**e**,**f**) and unpaired t-test (**b**); ****p<0.0001, ***p<0.001, **p<0.01, *p<0.05, ns, not significant, and mean±s.e.m. Each dot represents one individual’s reprogrammed HD-MSNs. The sample size (n) corresponds to the number of biological replicates.

Given the link between RCAN1 and TFEB activity, we asked if *RCAN1* KD would enhance autophagy function in HD-MSNs. We performed CYTO-ID assay (a fluorescence-based live-cell assay for accumulated autophagic vacuoles^10^), immunoblotting assay for p62/SQSTM1 expression (a marker widely used to monitor autophagic activity due to its binding to LC3 and degradation by autophagy^61^), and tandem monomeric mCherry-GFP-tagged LC3 (previously shown to distinguish pre-fusion autophagic compartments from mature acidic autolysosomes based on the differential pH sensitivity of GFP versus mCherry^62,63^). *RCAN1* KD increased CYTO-ID signal compared to shCtrl in HD-MSNs from multiple HD patients, which was reversed by overexpressing RCAN1 (Fig. 5a). The level of p62/SQSTM1 protein was also significantly decreased by *RCAN1* KD (Fig. 5b). Moreover, *RCAN1* KD increased the average number of both pre-fusion autophagosomes (mCherry-positive : GFP-positive) and post-fusion autolysosomes (mCherry-positive : GFP-negative) per cell, which was reversed by overexpressing RCAN1 (Fig. 5c).

We then assessed whether *RCAN1* KD would decrease HD-associated phenotype through TFEB. When HD-MSNs express TFEB cDNA, *RCAN1* KD mimicked the effect of TFEB phosphor-mutant (TFEB SA) which increased the average number of both pre-fusion autophagosomes (mCherry-positive : GFP-positive) and post-fusion autolysosomes (mCherry-positive : GFP-negative) per cell (Fig. 5d). Similarly, phospho-mutant of TFEB decreased Caspase 3/7 activation, Annexin V signal, and the formation of mHTT inclusion bodies which was replicated by *RCAN1* KD in the presence of wildtype TFEB (Fig. 5e-f). Altogether, these results indicate that *RCAN1* KD promotes HD-MSN resilience against degeneration largely through enhancing TFEB activity.

### Neuroprotection by G2 analog through reduction of RCAN1-CaN interaction

**Fig. 6 ∣:**
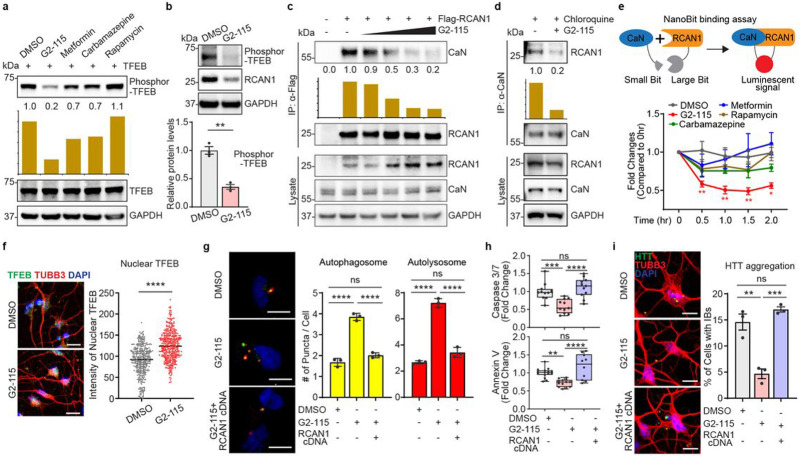
G2-115 promotes TFEB function by reducing RCAN1-CaN interaction and promoting TFEB nuclear localization. **a**, Immunoblotting analysis of autophagy inducer-treated fibroblasts with anti-phosphor-TFEB (Ser142) antibody. Cells were treated with DMSO, 0.5 μM of G2-115, 8 mM of metformin, 100 μM of carbamazepine, or 500 nM of rapamycin. **b**, Representative Immunoblotting (top) and quantification (bottom) of phosphor-TFEB (Ser142) in three independent HD-MSNs (HD.47, HD.40, HD.45, n=3) treated with DMSO or 0.5μM of G2-115. **c**, Immunoprecipitation analysis of Flag-RCAN1-transfected HEK293 cells with anti-Flag antibody followed by immunoblotting with anti-CaN antibody. Dose-response of cells was measured with 0.25, 0.5, 2.5, and 5 μM of G2-115. **d**, Immunoprecipitation analysis of chloroquine (lysosome inhibitor)-treated fibroblasts with anti-CaN followed by immunoblotting with anti-RCAN1 antibody. Cells were treated with DMSO or 0.5 μM of G2-115 and 60 μM of chloroquine (lysosome inhibitor), **e**, Experimental scheme of NanoBit binding assay (top). Binding assay of HEK293 cells transfected with RCAN1 fused to a large Bit and CaN fused to a small Bit. Cells were treated with autophagy inducers (2.0 μM of G2-115, 8 mM of metformin, 100 μM of carbamazepine, or 500 nM of rapamycin) (bottom), **f**, Representative image (left) and quantification (right) of nuclear TFEB in three independent HD-MSNs (HD.43, HD.45, HD.40, n=3) treated with DMSO or 0.5 μM of G2-115. Scale bars represent 20 μm. Each dot represents one reprogrammed cell positive for TFEB and DAPI. **g**, Representative images (left) of HD-MSNs expressing the tandem monomeric mCherry-GFP-LC3 reporter. Quantification (right) of autophagosome and autolysosome from cells having puncta from three independent HD-MSNs (HD.40, HD.47, HD.43, n=3) treated with DMSO or 0.5 μM of G2-115. Cells were transduced with Control or RCAN1 and measurements were performed in cells having puncta (from more than 50 cells per MSN line). Scale bars represent 20 μm. **h**, Quantification of caspase 3/7 activation (top) from three independent HD-MSNs (HD.40, HD.47, HD.45, n=11) and annexin V signal (bottom) from three independent HD-MSNs (HD.43, HD.47, HD.45, n=10~15) treated with DMSO or 0.5μM of G2-115. Cells were transduced with Control or RCAN1. **i**. Representative image (left) and quantification (right) of HTT inclusion bodies (IBs) in three independent HD-MSNs (HD.43, HD.40, HD.47, n=3) treated with DMSO or 0.5 μM of G2-115. Cells were transduced with Control or RCAN1. Cells were immunostained with anti-HTT and TUBB3 antibodies. An average of 300 cells per each were counted from three or more randomly chosen fields. Scale bars represent 10 μm. Statistical significance was determined using one-way ANOVA (**e**,**g**,**h**,**i**) and unpaired t-test (**b**,**f**); ****p<0.0001, ***p<0.001, **p<0.01, *p<0.05, ns, not significant, and mean±s.e.m. Each dot represents one individual’s reprogrammed HD-MSNs (**b**,**g**,**h**,**i**). The sample size (n) corresponds to the number of biological replicates (**b**,**f**,**g**,**h**,**i**).

We then wondered if the genetic effect of *RCAN1* KD on TFEB dephosphorylation for neuroprotection could be replicated by small molecules. We first tested various small molecules known to increase autophagy including G2-115, metformin, carbamazepine, and rapamycin^28,64,65^. Among the compounds tested, we found that G2-115 significantly reduced the phosphorylation of TFEB compared to other autophagy inducers (Fig. 6a). This effect was consistent in multiple HD-MSN lines in which G2-115 decreased the phosphorylation of endogenous TFEB (Fig. 6b). G2 was the original analog of glibenclamide identified as sufficient for the autophagic-enhancing activity which promoted degradation of misfolded α1-antitrypsin Z variant (ATZ) in mammalian cell models of α1-antitrypsin deficiency (ATD)^28,64^. Importantly, G2 analog, G2-115 was recently shown to reduce HD-MSN death and mHTT inclusion bodies in HD-MSNs^10^. Due to changes in TFEB phosphorylation, we tested whether G2-115 would affect the interaction between RCAN1 and CaN. Strikingly, G2-115 reduced the binding of RCAN1 to CaN in a dose-dependent manner when Flag-tagged RCAN1 was pulled down, and then CaN was probed with various concentrations of G2-115 (Fig. 6c). When endogenous CaN was pulled down in fibroblasts or HD-MSNs in the presence of lysosome inhibitor, Chloroquine (to keep the consistent level of RCAN1), G2-115 interrupted RCAN1-CaN interaction (Fig. 6d and Extended Data Fig. 6a). This effect was specific to G2-115 as other autophagy inducers did not affect RCAN1-CaN interaction (Extended Data Fig 6b). We also performed the NanoBit binding assay in HEK293 cells transfected with the interaction domain of RCAN1 (amino acid 89-197) and CaN (amino acid 1-391)^16^ fused with luciferase subunits which can generate luminescent signals when they interact. This assay confirmed the specificity of G2-115 in reducing RCAN1-CaN binding among other autophagy inducers, repeated at various concentrations of G2-115 (Fig. 6e and Extended Data Fig. 6c). Importantly, we tested whether G2-115 would affect the TFEB localization as its dephosphorylation would allow TFEB shuttling into the nucleus^26,58,59^. G2-115 significantly increased the nuclear localization of endogenous TFEB as determined in HD-MSNs from multiple patients (Fig. 6f). Altogether, our results indicate that G2-115 enhances TFEB functions by specifically reducing the interaction between RCAN1 and CaN.

To further test the rescuing effect of G2-115 potentially through reducing RCAN1 function, we first measured the autophagy activity by using tandem monomeric mCherry-GFP-tagged LC3 in multiple independent HD-MSNs. G2-115 increased the average number of both pre-fusion autophagosomes (mCherry-positive : GFP-positive) and post-fusion autolysosomes (mCherry-positive : GFP-negative) per cell, which was reversed by overexpressing RCAN1 (Fig. 6g). Additionally, neuronal cell death and the formation of HTT inclusion body were decreased by G2-115 and this effect was reversed by RCAN1 overexpression (Fig. 6h-i). Collectively, our results indicate that RCAN1-CaN is a chemically modifiable target that G2-115 can act on to increase neuronal resilience of HD-MSNs to neurodegeneration.

## Discussion

In the current study, we performed a comparative transcriptome analysis in longitudinally collected fibroblasts and corresponding reprogrammed MSNs derived from healthy individuals. MicroRNA-based direct fate conversion of adult fibroblasts to neurons has been shown to retain chronological age signature stored in starting fibroblasts^6^ and hallmarks of adult-onset HD pathologies are also captured in directly reprogrammed striatal MSNs from HD patient-derived fibroblast^9-11^. Therefore, directly reprogrammed human MSNs from longitudinally collected fibroblasts are the established human neuron platform that allows for studies of aging in human neurons in an isogenic background.

This analysis identified RCAN1 as an age-associated regulator whose expression was upregulated in aged-MSNs and human striatum. Mechanistically, we provide evidence that reducing RCAN1 function protects HD-MSNs by opening chromatin regions proximal to genes involved in longevity-regulating pathway. Knocking down *RCAN1* relieves its repression on its interactor CaN, which in turn dephosphorylates and promotes nuclear localization of TFEB and increased accessibility of chromatin regions that harbor TFEB binding sites. Moreover, we reveal the mechanism of the G2 analog that can mimic the protective effect of *RCAN1* KD by reducing the RCAN1-CaN interaction and phosphorylation of TFEB. Through this mode, the G2 analog can help clearance of mHTT inclusion bodies and the survival of HD-MSNs. Therefore, RCAN1 functions as an age-associated modifier that promotes neurodegeneration, and genetic or pharmacological intervention on RCAN1 activity can be potentially harnessed to promote neuronal resilience against the age-associated onset of neurodegeneration in HD.

Remarkably, reduced RCAN1 expression enhances chromatin accessibility of genes involved in longevity and autophagy to promote neuronal resilience against neurodegeneration in HD-MSNs. Interestingly, the increase in RCAN1 expression in the striatum of elderly individuals compared to young individuals was captured in reprogrammed MSNs from longitudinally collected fibroblasts. The increase in RCAN1 gene dosage is also implicated in Down syndrome as a gene in chromosome 21 trisomy, which increases the susceptibility to Alzheimer’s disease^20-24^. Future studies should be directed to further investigate the changes in RCAN1 expression in different ages of HD patient brains, or whether the changes in RCAN expression may be related to other neurodegenerative disorders.

Glibenclamide, a sulfonylurea drug has been used broadly in clinics as an oral hypoglycemic agent. A glibenclamide analog, G2, promoted autophagic degradation of misfolded α1-antitrypsin Z variant (ATZ) in mammalian cell models of α1-antitrypsin deficiency (ATD) disorder ^28,64^. We found a unique feature of a G2-115 as an autophagy inducer that promotes TFEB activity by reducing the RCAN1-CaN interaction to promote clearance of HTT inclusion bodies and neuronal survival. However, it remains unclear how G2-115 mechanistically interferes with RCAN1-CaN interaction. Previous studies provided the structural information of RCAN1-CaN binding that delineates the structural basis of RCAN1 and CaN interaction^16^. RCAN1 inhibits the activity of CaN directly by binding and blocking both substrates-binding sites and active site of CaN, in which RCAN1-CaN binding is disrupted when SPPASPP and TxxP motifs in the N-terminal domain of RCAN1 are mutated^16^. Our study was not designed to address whether G2-115 reduces the RCAN1-CaN interaction by interfering with RCAN1 stability through a secondary pathway or by binding directly to the interaction site in the N-terminal domain of RCAN1. Further investigations into the specific mechanism of how G2-115 reduces RCAN1-CaN interaction may provide new insights into how small compounds can be used to increase the resilience against neurodegeneration in HD. Additionally, screening additional small molecules that directly interfere with the interaction between RCAN1 and CaN, and replicating the neuroprotective effect of RCAN1 inhibition may offer a new therapeutic target which may alleviate neurodegeneration in HD.

## Methods

### Plasmids, shRNAs, and Cell lines.

The construction of all plasmids used for MSNs reprogramming in this study has been previously described^6,9-11,38,66-70^, and they are publicly available at Addgene as pTight-9-124-BclxL (#60857), rtTA-N144 (#66810), pmCTIP2-N106 (#66808), phDLX1-N174 (#60859), phDLX2-N174 (#60860), and phMYT1L-N174 (#66809). For the overexpression of RCAN1, the RCAN1 genomic sequence was cloned and ligated into the pcDNA, pcDNA-Flag-HA, and N174-lentiviral vector. For the overexpression of TFEB wildtype and SA (S142/211A), pcDNA3.1-TFEB-WT-MYC (#99955) was obtained from Addgene, mutagenized, and ligated into the N174-lentiviral vector. Lentiviral shRNA control (shCtrl) (SHC002), human RCAN1 shRNAs (TRCN0000256296), and human PPPC3A (CaN) shRNA (TRCN0000342619) were obtained from Sigma. To visualize free autophagosomes and autolysosomes, FUW mCherry-GFP-LC3 (# 110060) was obtained from Addgene. Adult dermal fibroblasts from symptomatic HD patients (Coriell NINDS and NIGMS Repositories: ND33947, ND30013, GM02173, GM04230, GM04198, GM4194), presymptomatic HD patients (Coriell NINDS and NIGMS Repositories: GM04717, GM04861, GM04857, GM04855, GM04829), healthy control (Coriell NINDS and NIGMS Repositories: AG03440, AG0495, AG11732, AG10047, AG12956, AG02187, GM02171) and longitudinal healthy individuals (Coriell NINDS and NIGMS Repositories: AG10049, AG16030, AG10047, AG14048, AG04456, AG14251) were acquired from the Coriell Institute for Medical Research.

### Antibodies.

Primary antibodies used for immunostaining and immunoblot included rabbit anti-MAP2 (CST, #4542), rabbit anti-DARPP-32 (19A3) (CST, #2306), rabbit anti-GABA (Sigma-Aldrich, A2052), mouse anti-NCAM1 (ERIC) (Santa Cruz, sc-106), rabbit anti-NEUN (Millipore, ABN78), mouse anti-ACTL6B (Antibodies Incorporated, 75-311), mouse anti-tubulin β III (Covance, MMS-435P), rabbit anti-tubulin β III (Covance, PRB-435P-100), rabbit anti-RCAN1/DSCR1 (Sigma-Aldrich, D6694), rabbit anti-pan-Calcineurin A (Cell signaling, 2614), rabbit anti-TFEB (Cell signaling, 4240), rabbit anti-phosphor-TFEB (Ser142) (Millipore, ABE1971), rabbit anti-phosphor-TFEB (Ser211) (Cell signaling, 37681), rabbit anti-p62/SQSTM1 (Abcam, ab109012), mouse anti-Flag (Sigma-Aldrich, F1804), rabbit anti-GAPDH (Santa Cruz, sc-32233) antibodies. The secondary antibodies included goat anti-mouse, rabbit IgG (H+L) HRP secondary antibody, and goat anti-rabbit, mouse, rat, or chicken IgG conjugated with Alexa-488, −594, or −647 (Thermo Fisher Scientific).

### Primary cell culture.

Adult human fibroblasts were cultured in fibroblast media (FM) comprised of Dulbecco’s Modified Eagle Medium (DMEM) (high glucose and no glutamine) supplemented with 15% fetal bovine serum (FBS) (Gibco), 0.01% β-mercaptoethanol, 1% non-essential amino acids, 1% sodium pyruvate, 1% GlutaMAX, 1% 1M HEPES buffer solution, and 1% penicillin/streptomycin solution (all from Invitrogen). Cells were only maintained for up to 15 passages.

### Lentiviral preparation.

Lentiviral production was carried out separately for each plasmid, but they were transduced together as a single cocktail as previously described^10,67^. Briefly, the supernatant was collected 72 h after transfection of Lenti-X^™^ 293T Cell Line (Clonetech) with each plasmid, in addition to psPAX2 and pMD2.G, using polyethyleneimine (PEI, Polyscience). Collected lentiviruses were filtered through 0.45 μm PES membranes, mixed for a single cocktail, and incubated with the Lenti-X concentrator overnight to concentrate the virus to 10-fold. Concentrated lentiviruses are resuspended in 1/10 of the original volume with 1 x PBS after spinning down at 1,500 g for 45 min at 4 °C. In centrifuge tubes, add 7 ml of 20 % sucrose cushion solution (20 % Sucrose, 100 mM NaCl, 20 mM HEPES (pH 7.4), 1 mM EDTA in distilled water), and then overlay the resuspended lentiviruses on the sucrose solution. After centrifugation at 70,000 g for 2 h at 4 °C, viral pellets were resuspended in 10 % sucrose solution (10 % Sucrose, 25 mM HEPES (pH 7.3) in DPBS) and stored at −80 °C. Typical titers of lentivirus range from 1x10^7^ to 2.5x10^8^ infection-forming units per milliliter (IFU/ml).

### MSNs reprogramming.

Direct neuronal reprogramming of human fibroblasts to MSNs was performed as previously described^6,9-11,38,66-70^. Briefly, human fibroblasts were seeded onto Costar 6-well cell culture vessels (Corning) at a density of 300,000 cells/well. The following day, each plate was transduced with the lentiviral cocktail of pTight-9/9*-124-BclxL, rtTA, CTIP2, DLX1, DLX2, and MYT1L in the presence of polybrene (8 ug/mL, Sigma-Aldrich). 4 mL of the lentiviral cocktail / fibroblast medium (FM) was added to each well then spinfected at 1,000 g for 30 min at 37 °C using a swinging bucket rotor. One day post-transduction (PID 1), cells were washed with PBS and added fresh FM (2 mL/well) supplemented with doxycycline (Dox, 1 μg/mL) (Sigma-Aldrich). After 2 days (PID 3), the medium was changed to fresh FM supplemented with Dox and puromycin (3 μg/ml) (Life Technologies). After 2 days (PID 5), cells were replated onto polyornithine/laminin/fibronectin-coated glass coverslips previously treated with nitric acid and added with FM supplemented with Dox (1 μg/ml). The following day (PID 6), media was then changed to Neurobasal^™^-A Medium (Gibco, Cat# 10888022) containing B-27^™^ Plus Supplement (Gibco, Cat# A3582801) and GlutaMAX Supplement (Gibco, Cat# 35050061) supplemented with Dox (1 μg/ml), valproic acid (1 mM), dibutyryl cAMP (200 μM), BDNF (10 ng/ml), NT-3 (10 ng/ml), Retinoic Acid (1 μM), RevitaCell Supplement (RVC, 1x), ascorbic acid (200 μM), and antibiotics (puromycin, 3 μg/ml; blasticidin, 3 μg/ml; geneticin, 300 μg/ml). Dox was replenished every two days and half-volume medium changes were performed every 4 days. At PID 14, media was switched to Brainphys (Stemcell, Cat# 05793) containing NeuroCult SM1 neuronal supplement and N2 supplement-A supplemented with Dox (1 μg/ml), valproic acid (1 mM), dibutyryl cAMP (200 μM), BDNF (10 ng/ml), NT-3 (10 ng/ml), Retinoic Acid (1 μM), RevitaCell Supplement (RVC, 1x), ascorbic acid (200 μM), and puromycin (3 ug/ml). The addition of Blasticidin and geneticin was halted after PID 10 and puromycin was continuously added until PID 30. The addition of RVC and ascorbic acid was also terminated after PID 21.

### Reduction-of-function testing of GeM-HD modifiers.

To streamline the modifier gene identification, we adapted 96-well culture plates to be assayed for neuronal death using Sytox-Green as a cell death marker as previously described^9^ (approximately 3000 cells counted in each well). For this assay, we used HD-MSN from the GM04194 line (CAG repeat size 46; HD.46) which showed the two-fold increase in cell death at around 50% compared to Control (Ctrl)-MSNs from the healthy individual (GM02171). We carried out individual KD of 246 genes selected among 308 suggested modifier genes based on their expression level in HD-MSN transcriptome^9^ (Supplementary Table 1). We performed reduction-of-function testing by adding lentivirus carrying gene-specific shRNAs to HD-MSN at post-induction day 21 (PID 21), a time point when miRNA-induced cells start acquiring the neuronal identity^8^, to avoid interference with early stages of neuronal reprogramming. Cells were then cultured to reprogramming day 35 (PID 35), a time point when HD-MSNs start undergoing spontaneous neuronal death compared to control-MSNs^9^, and the average cell death level for each gene KD was compared to the average level detected with scrambled control shRNA (shCtrl).

### Sytox assay in live cells.

0.1 μM Sytox gene nucleic acid stain and 1 μl/mL of Hoechst 33342 Solution were added into the cell medium. Samples were incubated for at least 15 mins at 37 °C before imaging. Images were taken using Leica DMI 4000B inverted microscope with Leica Application Suite (LAS) Advanced Fluorescence.

### Apoptosis assay in live cells.

Cells were treated with 1X Essen Bioscience IncuCyte^®^ Caspase-3/7 Green Reagent (final concentration 5 μM) and 1X Essen Bioscience IncuCyte^®^ Annexin V Green or Red Reagent at PID 22 or 26. Image scheduling, collection, and analysis were conducted with the IncuCyte^®^ S3 LiveCell Analysis System and IncuCyte S3 v2017A software. Treated plates were imaged every two hours for 7 days. At each time point, over 2 images were taken per well in brightfield, FITC, and TRITC channels. Images were analyzed for the number of green or red objects per well. For the apoptotic index, the number of green or red objects (i.e., fluorescence cells) divided by phase area (μm^2^) per well was quantified by the IncuCyte^®^ S3 Live-Cell Analysis System.

### Immunostaining analysis.

Cells were fixed with 4% paraformaldehyde for 20 min at room temperature (RT) and then permeabilized with PBS containing 0.2% Triton X-100 for 10 min at RT. Cells were then blocked with blocking buffer (5% BSA and 1% goat serum in PBS) for 1 h at RT. Primary antibodies were incubated in blocking buffer at 4 °C overnight. Cells were washed with PBS for 5 min three times and then incubated with secondary antibodies in blocking buffer for 1 h at RT. Cells were washed with PBS two times and incubated with DAPI for 10 min. Images were captured using a Leica SP5X white light laser confocal system with Leica Application Suite (LAS) Advanced Fluorescence 2.7.3.9723.

### Immunoblot analysis.

Cells were lysed with RIPA buffer containing 1 x protease inhibitor /1 x phosphatase inhibitor or SDS buffer (2 % SDS, 10 % Glycerol, 12.5 mM EDTA, 50 mM Tris-HCL pH 6.8). The concentrations of whole-cell lysates were measured using the Pierce BCA protein assay kit. Equal amounts of whole-cell lysates were resolved by SDS-PAGE and transferred to a nitrocellulose membrane (GE Healthcare Life Sciences, #10600006) using a transfer apparatus according to the manufacturer’s protocols (Bio-Rad). After incubation with blocking buffer (5 % BSA, 0.1 % Tween-20 in TBS) for 1 h, the membrane was incubated with primary antibodies at 4°C overnight. After washing with TBS-T (0.1 % Tween-20 in TBS) three times for 5 min, the membrane was then incubated with a horseradish peroxidase-conjugated secondary antibody for 30 min at RT. The membrane was washed with TBS-T three times for 10 min and developed with the ECL system (Thermo Scientific, #34580) according to the manufacturer’s protocols.

### RNA preparation and RT-qPCR.

Total RNA was extracted using RNeasy Micro Kit (Qiagen) and reverse transcription was performed using the Superscript IV first strand synthesis system for RT-PCR (Invitrogen) according to the manufacturer’s protocol. Quantitative PCR was performed using SYBR Green PCR master mix (Applied Biosystems) and StepOnePlus Real-Time PCR system (Applied Biosystems, 4376600) according to the manufacturer’s protocol against target genes. Quantitative PCR analysis was done with the following primers: RCAN1; 5’-TGGAGCTTCATTGACTGCGA-3’ and 5’-CTCAAATTTGGCCCGGCAC-3’, PPPC3A; 5’-GCGCATCTTATGAAGGAGGGA-3’ and 5’-TGACTGGCGCATCAATATCCA-3’, GAPDH; 5’-ATGTTCGTCATGGGTGTGAA-3’ and 5’-TGTGGTCATGAGTCCTTCCA-3’.

### Omni-ATAC-sequencing preparation.

Omni-ATAC was performed as outlined in Corces et al^37^. Briefly, each sample was treated with DNase for 30 minutes before collection. Approximately 50,000 cells were collected for library preparation. Transposition reaction was completed with Nextera Tn5 Transposase (Illumina Tagment DNA Enzyme and Buffer Kit, Illumina) for 30 minutes at 37 °C, and library fragments were amplified under optimal amplification conditions. Final libraries were purified by the DNA Clean & Concentrator 5 Kit (Zymo, USA). Libraries were sequenced on Illumina NovaSeq S4 XP (Genome Technology Access Center at Washington University in St. Louis).

### ATAC-Seq analysis.

For ATAC-seq analysis in directly reprogrammed neurons, the raw data containing FASTQ files were uploaded to Partek Flow^®^ Software (Partek Incorporated, St. Louis, Missouri, United States). ATAC-seq reads were aligned to hg38 human genome assembly using BWA, and uniquely mapped reads were used for downstream analysis. Differential peaks were identified using Partek’s Gene Specific Analysis (GSA) algorithm with a cut-off of fold-change (FC) ≥ 1.5 and FDR < 0.05 and regarded as peaks gained or lost. Gained peaks in shCtrl-HD-MSNs were combined and defined as open (more accessible) chromatin regions. Conversely, all reduced peaks in shCtrl-HD-MSNs were defined as closed chromatin regions.

### mCherry-GFP-LC3 quantification.

FUW mCherry-GFP-LC3 was a gift from Anne Brunet (Addgene plasmid #110060; http://n2t.net/addgene: 110060; RRID: Addgene_110060). The concentrated lentivirus of mCherry-GFP-LC3 was added to reprogrammed MSNs at PID 20. For imaging of cells expressing mCherry-GFP-LC3, cells were washed once with PBS, fixed, and stained by anti-TUBB3 antibody at PID 26, after validation of the expression of GFP and mCherry by microscopy. Images were captured using a Leica SP5X white light laser confocal system with Leica Application Suite (LAS) Advanced Fluorescence 2.7.3.9723.

### Protein binding assay.

The NanoBiT Protein:Protein Interaction system (Promega, #N2014) was used for the binding assay of RCAN1-CaN interaction according to the manufacturer’s protocol. HEK293 cells plated in a 96-well plate were transfected with 25 ng (per well) of pBiT1.1-N-RCAN1 (89-197) and pBiT2.1-C-CaN (1-391) using PEI (Polysciences, 24765) with Opti-MEM (Life Technologies, 31985). Forty-eight hours after transfection, 25 μL of Nano-Glo live Cell Assay reagent was added to each well after autophagy inducers were treated. After the initial measurement, luminescence values were measured every 30 minutes using the Synergy H1 Hybrid plate reader (BioTek).

### Statistical analysis.

Statistical analysis was performed in GraphPad Prism v9.1 (GraphPad Software). Data are expressed as mean±s.e.m from at least three independent experiments unless otherwise indicated. Statistical comparisons were performed by an unpaired t-test with a two-tailed distribution or one-way ANOVA with a Bonferroni post-test using Prism 6.0 (GraphPad Software Inc.). Statistical significance was set at p<0.05, with the following standard abbreviations used to reference *P* values: ns, not significant; *p<0.05; **p<0.01; ***p<0.001; ****p<0.0001. Detailed statistical information for each experiment is provided in the corresponding figure legend.

## Extended Data

**Extended Data Fig. 1 ∣:** Gene expression profiling in longitudinally collected fibroblasts and corresponding reprogrammed MSNs **a**, Information of fibroblast samples used in the longitudinal study. **b,** RT-qPCR analysis of DARPP-32 expression in longitudinally aged MSNs. Statistical significance was determined using t-test; ***p<0.001, **p<0.01, and mean±s.e.m. **c** and **d**, Heatmap of Differentially Expressed Genes (DEGs) in fibroblasts (**c**) and MSNs (**d**) (FDR<0.05, ∣FC∣≥1.5). **e**, Venn diagram of the genes enriched in calcium signaling pathway from old HD-MSNs.

**Extended Data Fig. 2 ∣:** Age-associated genes in longitudinally aged MSNs **a**, Upstream regulator analysis of up- or down-regulated genes in old fibroblasts and MSNs. **b**, Gene network of upstream regulators and DEGs. **c**, Representative immunoblotting (top) and quantification (bottom) of RCAN1 expression in six MSNs aged 22, 29, 24 (young) and 53, 50, 60 (old) years old (n=6). **d**, Quantification of RCAN1 mRNA from six longitudinal individuals (I, II, and III) (n=12). Statistical significance was determined using one-way ANOVA (**c**) and t-test (**d**). **p<0.01, ns, not significant, and mean±s.e.m.

**Extended Data Fig. 3 ∣:** Identification of modifier genes whose reduction protects HD-MSNs from degeneration **a**, Experimental scheme of testing of genetic modifiers in HD-MSNs reprogrammed from symptomatic HD patient-derived fibroblasts. **b**, Representative images (left) and quantification (right) of MAP2-, NCAM-, NEUN-, ACTL6B-, DARPP-32-, and GABA-positive cells from four independent reprogrammed HD-MSNs (HD.43, HD.40, HD.47, HD.45, n=4). Corresponding fibroblasts were used as a negative control for each staining. An average of 300 cells per each were counted from three or more randomly chosen fields. Scale bars represent 20 μm. **c**, High-content imaging of Sytox green dye accumulation in reprogrammed HD-MSNs (HD.46) in a 96-well format. Representative images of reprogrammed HD-MSNs in each well of a 96-well plate, immunostained with anti-GABA, TUBB3, and DARPP-32 antibodies (left). Example pictures for high content image analysis to measure cell death levels in HD-MSNs (right). Hoechst for counting the whole cell population and Sytox-green for marking dead cells. **d**, Quantification of Sytox-positive cells over total Hoechst-positive cells from MSNs at post-induction day 35 (PID 35). MSNs derived from symptomatic HD patient (HD.46) and healthy controls (Ctrl.17) (n=2, biological replicates). **e**, Quantification of Sytox-positive cells in HD-MSNs (HD.46) transduced with shRNAs of modifier genes. The genes corresponding to shRNAs that significantly lowered cell death levels were marked (red) within the pink area (+/− 10 % of cell death level from healthy control) compared to the average scrambled control shRNA. Statistical significance was determined using unpaired t-test and mean±s.e.m (n=2 biological replicates); RCAN1 (p=0.0143), RTCA (p=0.0198), and UBE2D4 (p=0.0073). **f**, Additional validation of the identified HD-modifier genes whose KD rescues HD-MSNs from neuronal death in multiple patient lines. Representative image (left) and quantification (right) of Sytox-positive cells from three independent HD-MSNs (HD.46, HD.44, HD.43, n=12 independent reprogramming) transduced with shRNAs of each gene. Scale bars represent 100 μm. **g**, Representative image (left) and quantification (right) of cells with HTT inclusion bodies (IBs) in HD-MSNs transduced with shRNAs of each gene. Cells were immunostained with anti-HTT and TUBB3 antibodies. An average of 100 cells per each were counted from four to six randomly chosen fields of HD-MSNs (HD.40). Scale bars represent 10μm. Statistical significance was determined using unpaired t-test (**d**,**e**) and one-way ANOVA (**f**,**g**); ****p<0.0001, *p<0.05, ns, not significant, and mean±s.e.m. Each dot represents one individual’s reprogrammed HD-MSNs (**b**,**d**,**e**,**f**). The sample size (n) corresponds to the number of biological replicates (**b**,**d**,**e**,**f**).

**Extended Data Fig. 4 ∣:** Validation of reprogrammed neurons of rescuing or non-rescuing condition for ATAC-sequencing **a**, RCAN1 expression in fibroblasts transduced with shRCAN1 (top) or RCAN1 (middle) in a dose-dependent manner. RCAN1 expression in HD-MSNs (HD.43) transduced with shCtrl, shRCAN1, or RCAN1 (bottom). **b**, Representative image (top) and quantification (bottom) of DARPP-32-positive cells from four independent HD-MSNs transduced with shCtrl, shRCAN1, or shCaN (HD.43, HD.40, HD.47, HD.45, n=4). Cells were immunostained with anti-DARPP-32 and TUBB3 antibodies. An average of 183 cells of each were counted from three or more randomly chosen fields. Scale bars represent 10 μM. **c**, RT-qPCR analysis of the expression of RCAN1 and CaN in (**b**) (n=4). Statistical significance was determined using VA (**b**) and unpaired t-test (**c**); ****p<0.0001, ns, not significant, and mean±s.e.m. The sample size (n) corresponds to the number of biological replicates.

**Extended Data Fig. 5 ∣:** Validation of phosphor-mutant of TFEB **a**, Expression of phosphor-TFEB in fibroblasts transduced with Control, TFEB wildtype, or phosphor-mutant (S142/211A).

**Extended Data Fig. 6 ∣:** Neuroprotective role of G2-115 through reducing RCAN1-CaN interaction **a**, Immunoprecipitation analysis of RCAN1-transduced fibroblasts with anti-CaN antibody followed by immunoblotting with anti-RCAN1 antibody. Cells are treated with 0.5 μM of G2-115 and 60 μM of chloroquine (lysosome inhibitor). **b**, Immunoprecipitation analysis of RCAN1-transduced fibroblasts with anti-CaN followed by immunoblotting with anti-RCAN1 antibody. Cells were treated with DMSO or 0.5 μM of G2-115, 8 mM of metformin, and 500 nM of rapamycin. **c**, Experimental scheme of NanoBit binding assay (top). Binding assay of HEK293 cells transfected with RCAN1 fused to large Bit and CaN fused to small Bit. Cells were treated with 0.5, 1.0, 1.5, and 2.0 μM of G2-115 in a dose-dependent manner (bottom). Statistical significance was determined using one-way ANOVA (c); **p<0.01, *p<0.05, and mean±s.e.m.
